# A new role for an old enemy

**DOI:** 10.7554/eLife.06424

**Published:** 2015-02-10

**Authors:** Richard M Monaghan, Alan J Whitmarsh

**Affiliations:** Faculty of Life Sciences, University of Manchester, Manchester, United Kingdom; Faculty of Life Sciences, University of Manchester, Manchester, United Kingdom, alan.j.whitmarsh@manchester.ac.uk

**Keywords:** AKT, cell survival, kinase-independent, PI3K, oncogenic, kinase inhibitor, human, Mouse

## Abstract

Drugs that change the shape of AKT, a protein kinase that promotes tumor growth, may be more effective than drugs that only target its enzymatic activity.

**Related research article** Vivanco I, Chen ZC, Tanos B, Oldrini B, Hsieh WY, Yannuzzi N, Campos C, Mellinghoff IK. 2014. A kinase-independent function of AKT promotes cancer cell survival. *eLife* **3**:e03751. doi: 10.7554/eLife.03751

**Image** The effect of AKT on cancer cells is more complex than previously thought

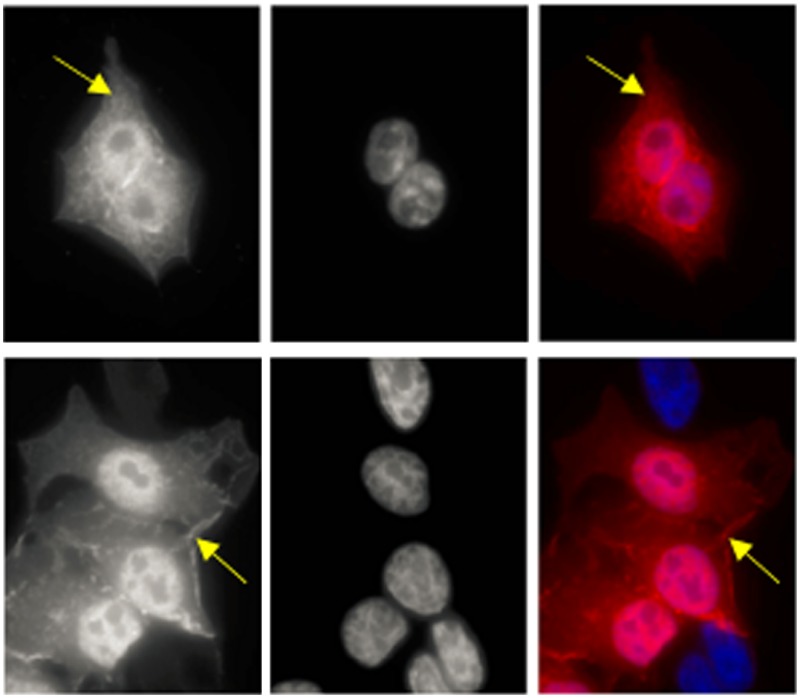

Many human cancers rely on the abnormal activation of a protein called AKT to promote the growth and survival of tumors. AKT, which is also known as protein kinase B, is therefore a potential target for anti-cancer therapies. Several compounds that inhibit the kinase activity of AKT have been identified, but progressing these into the clinic requires a more complete understanding of their effect on AKT in cancer cells (Fang et al., 2013; Dienstmann et al., 2014).

Now, in *eLife*, Ingo Mellinghoff of the Memorial Sloan-Kettering Cancer Center (MSKCC) and colleagues—including Igor Vivanco and Zhi Chen as joint first authors—address this issue by examining how two different types of AKT inhibitor regulate the survival of cancer cells. This revealed a previously unrecognized role for AKT that is independent of its role as a kinase (Vivanco et al., 2014).

AKT is a component of a signaling network within cells that responds to signals that promote cell growth and survival. It is activated by another protein called PI3K that generates specific lipid molecules to recruit AKT to the cell membrane (Figure 1). In human cancers this signaling network is frequently disturbed by mutations that cause PI3K and/or AKT to be more active (Fruman and Rommel, 2014).

**Figure 1.**
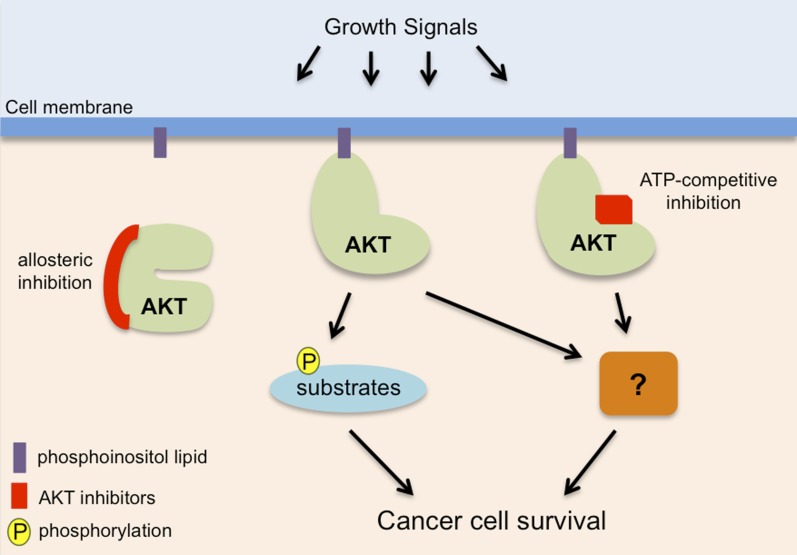
AKT, also known as protein kinase B, promotes cancer cell survival in two distinct ways. AKT (pale green) is recruited to phosphoinositol lipids (purple) at the cell membrane. Normally it is only activated in response to growth or survival signals, but it has increased activity in many cancers. It has been known for some time that AKT promotes the survival of cancer cells by adding phosphate groups (yellow) to protein substrates (light blue): this process involves ATP (not shown) binding to an active site in the kinase domain of the AKT, so it can be inhibited by drugs that compete with ATP to bind to this site (red rectangle). Vivanco, Chen et al. show that AKT can also promote cancer cell survival in a way that is independent of its kinase function: however, the details of this process remain unclear (hence the question mark). Drugs that compete with ATP do not inhibit this kinase-independent role, but allosteric inhibitors (left; see main text) inhibit both the kinase-dependent and kinase-independent roles of AKT, so they have the potential to be more effective therapies to treat cancer.

Some inhibitors work by binding to the active site of the enzyme domain within the AKT protein: this prevents ATP binding to the active site and thus inhibits the kinase activity of AKT. Unfortunately the clinical use of inhibitors that work by competing with ATP binding has been limited due to their toxic side effects, which may be due to them also targeting other kinases. Other inhibitors work by altering the shape or ‘conformation’ of AKT in a process known as allosteric inhibition (Fang et al., 2013; Dienstmann et al., 2014). An allosteric inhibitor that works by stabilizing AKT in an inactive state—which prevents ATP or other proteins binding to the active site—is currently in Phase II clinical trials.

The study by Vivanco, Chen et al.—who are based at MSKCC, the Spanish National Cancer Research Center and Cornell University—found that this allosteric inhibitor was better at killing cancer cells than inhibitors that compete with ATP binding. This suggested that AKT has an additional role in the survival of cancer cells that is independent of its kinase activity. Further support for this idea came from experiments demonstrating that a mutant of AKT with no enzyme activity could protect cancer cells from death.

Although the details of this second role are not clear, Vivanco, Chen et al. show that another domain in AKT called the PH domain is important. PH domains in other proteins generally bind to lipid molecules, but these latest results suggest that the PH domain in AKT may promote cancer cell survival independently of its ability to bind to lipids. Identifying molecules that can bind to kinase-inactive AKT mutants would help to resolve this issue.

Is the kinase-independent role of AKT in promoting cell survival a feature of human cancers? Vivanco, Chen et al. characterized a mutant of AKT with no kinase activity that was originally found in a sample of skin cancer cells. When this mutant version was expressed in skin cells it promoted their survival. Furthermore, they found that the most common mutation in AKT present in cancer cells—which promotes the interaction of AKT with the cell membrane—also promotes cell survival independent of kinase activity. These findings suggest that the kinase-independent role of AKT could have widespread importance in tumor formation.

Other examples of protein kinases that have enzyme independent roles in promoting tumor formation have been reported (e.g., Gustafson et al., 2014; Holderfield et al., 2014; Tan et al., 2015), suggesting that this is a common characteristic. It is predicted that up to 10% of protein kinases found in humans lack kinase activity. These ‘pseudokinases’ are likely to have similar roles to the kinase-independent roles of true protein kinases (Zhang et al., 2012).

The study by Vivanco, Chen et al. highlights the importance of fully appreciating the non-enzymatic roles of proteins in both healthy and diseased cells. It suggests that allosteric inhibitors of AKT are likely to be more useful for treating AKT-dependent cancers than those that specifically target the kinase domain. More generally, it suggests that finding allosteric inhibitors of other protein kinases linked with human diseases may prove to be a fruitful approach to use to develop new therapies.
